# Noninvasive ventilation in acute respiratory failure due to H1N1 influenza

**DOI:** 10.4103/0970-2113.76301

**Published:** 2011

**Authors:** Prasanta R. Mohapatra, Naveen Dutt, Sushant Khanduri, Baijayantimala Mishra, Ashok K. Janmeja

**Affiliations:** *Department of Pulmonary Medicine, Government Medical College and Hospital, Chandigarh, India*; 1 *Department of Virology, Postgraduate Institute of Medical Education and Research, Chandigarh, India*

**Keywords:** ARDS, influenza A virus-H1N1 subtype, noninvasive ventilation

## Abstract

We present a case of severe H1N1 influenza with hypoxemic acute respiratory failure necessitating mechanical ventilation benefited from noninvasive positive pressure ventilation (NIPPV). The NIPPV may be of great use in treating patients with H1N1-related acute respiratory distress syndrome in a resource poor setting or when invasive ventilator is unavailable.

## INTRODUCTION

Novel H1N1 influenza virus spread rapidly led to a worldwide pandemic. During the pandemic, a significant number of patients became critically ill primarily because of respiratory failure. Most of these patients required intubation and mechanical ventilation. Need and care of such patients have overwhelmed the already-stretched intensive care unit (ICU) at places. We discuss the management issues of a case of severe H1N1 influenza with hypoxemic acute respiratory failure necessitating mechanical ventilation benefited from noninvasive positive pressure ventilation (NIPPV).

## CASE REPORT

**Figure 1:**
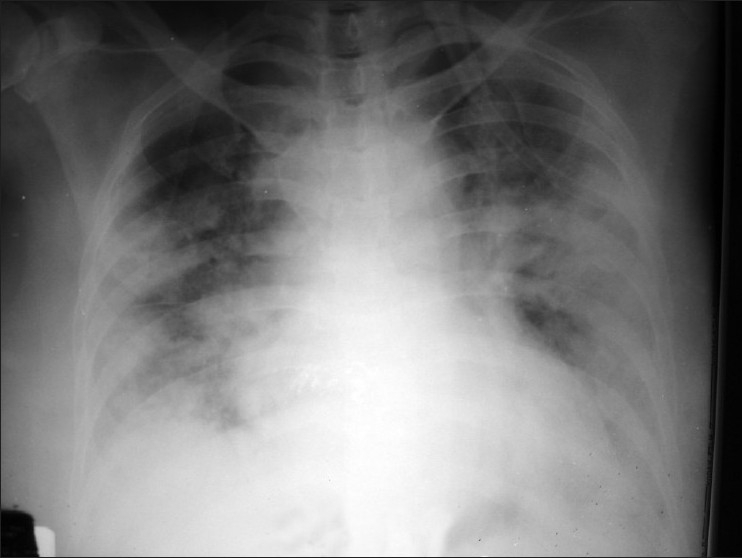
Chest radiograph showing ill-defined bilateral air-space opacities

A 65-year-old female presented to our emergency room on 22nd December 2009 with complaints of fever, dry cough, and progressive shortness of breath over 3 days. Her illness was progressively worsened over a day. She was admitted as a suspected case of severe H1N1 influenza. On admission, the patient had elevated temperature (39.6°C), respiratory rate 46/min, pulse rate 130/min, arterial blood pressure 100/60 mmHg, oxygen (O_2_) saturation 60% on room air. She was immediately given supplemental oxygenation (50% O_2_ by ventimask). Physical examination of chest revealed bilateral crackles in infrascapular area. A chest radiograph showed extensive bilateral opacities [Figure 1]. Influenza-A pneumonia was suspected and she was given oral oseltamivir (75 mg twice daily for 5 days), parenteral co-amoxyclav and azithromycin along with systemic corticosteroid. The nasopharyngeal swab specimen was positive for Influenza-A (H1N1) virus, using real-time reverse transcription-polymerase chain reaction (rRT-PCR) tested in the Virology Department of Postgraduate Institute of Medical Education and Research, Chandigarh. Laboratory investigations revealed total leukocyte count of 13,400 (86% neutrophils) and platelet count 2,80,000/mm^3^. Serum electrolytes, liver, and renal function tests were normal. Blood sugar was 90 mg/dl. Arterial blood gases at presentation were PO_2_, 49.5; PCO_2_, 33.8; bicarbonate, 17.2; pH, 7.32; O_2_ sat, 78.3% on 50% O_2_ on ventimask. The patient had developed acute respiratory distress syndrome (ARDS) rapidly. The patient needed invasive ventilatory support immediately, but could not be provided due to logistic reasons. A trial of noninvasive ventilation was given to the patient and she was continued on NIPPV with O_2_ and ABG was repeated every 6 h which showed improvement. Chest radiograph showed further deterioration with extensive opacity bilaterally.

**Figure 2:**
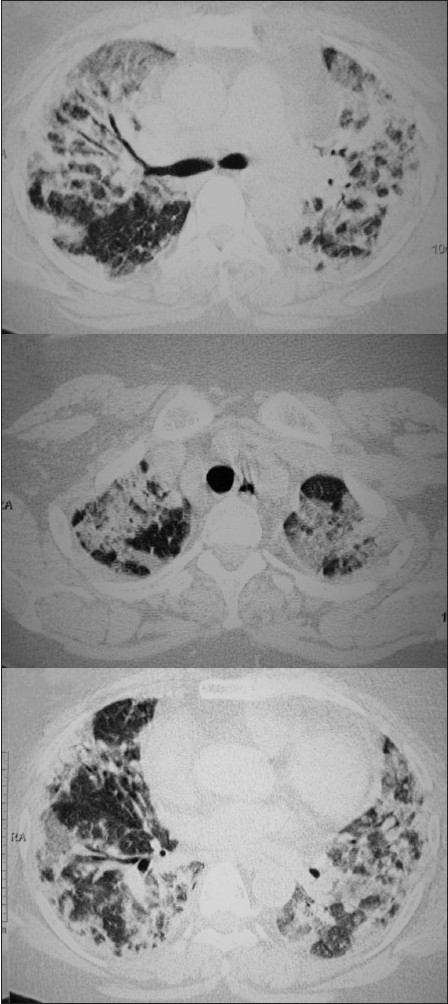
Lung windows of CT thorax showing bilateral multifocal consolidation and air bronchogram

The NIPPV was administered continuously for next 3 days through a facial mask with O_2_ support (10 cmH_2_ O maximum inspiratory airway pressure and a 5 cmH_2_O expiratory pressure) and she was maintaining the saturation of 88–90%. After 3 days, NIPPV was administered intermittently. Patient started maintaining SpO_2_ of 90% on 50% FiO_2_. Arterial oxygenation improved slowly with PaO_2_/FiO_2_ ratio improved subsequently over 6 days. There was no left heart dysfunction on echocardiogram. The patient had no other comorbidity. A CT thorax was done during the phases of recovery about 2 weeks after development of illness, demonstrated bilateral ground-glass opacities [Figure 2].

## DISCUSSION

The NIPPV is of great use in the management of hypoxemic respiratory failure due to chronic obstructive pulmonary disease (COPD); however, its role in ARDS secondary to severe pneumonia is still controversial. Only one case report of H1N1 influenza pneumonia managed by NIPPV has been reported.[1] Among the available reports even with invasive ventilator, the mortality rate reached nearly 60% for H1N1-related ARDS.[2]

Analysis of the first randomized trial of NIPPV in 56 patients with pneumonia revealed that only the subgroup of patients who had concomitant COPD appeared to benefit from NIPPV.[3] In another study by Rocker *et al*.,[4] there was a 66% success rate when NIPPV was used as the initial mode of assisted ventilation in 10 patients with ALI/ARDS. One recent study support the use of NIPPV is associated with reduction of endotracheal intubation and mortality rates in ARDS.[5] Initially, we could not provide invasive ventilator support in our patient. Later, when ventilator was available we did not intubate and shift to invasive ventilator as she had shown a significant improvement in maintaining O_2_ saturation. Her respiratory rates decreased at the end of the first hour, and thereafter remained stable. The extent of involvement and damage to lung parenchyma is evident by the CT scan of our patient. One recent and large study (105 patients) that addressed the success of NIPPV in severe pneumonia found that the use of NIPPV compared with high concentration O_2_ therapy decreased the need for intubation, the incidence of septic shock, and ICU mortality, even though the seven patients with ARDS had a poor outcome.[6] Current evidence therefore suggests as in our case that NIPPV may be reasonable in some patients of ARDS due to severe H1N1 pneumonia not responding to standard medical and O_2_ support. However, as the intubation rates are high, NIPPV should ideally be used in patients with an invasive mechanical ventilator on standby. It is the subgroup of patients with relatively mild, early ARDS who are likely to benefit from NIPPV and avoid intubation.

The adding NIPPV to standard therapy is associated with lower rates of endotracheal intubation and decreased mortality. During SARS epidemic, the best response was obtained in 60 patients with noninvasive ventilation for acute hypoxic failure.[7] There a potential for the H1N1 virus to cause severe acute respiratory failure necessitating mechanical ventilation in a significant number of the population. The hospitals today are frequently facing difficulties in stretching their limited resources such as ventilators and beds in ICUs.[8,9] The peak incidence of H1N1 pneumonia during late 2009 demanded the need of mechanical ventilators and intensive care beds out of proportion to the resources available in our hospital. This case reminds us the success of NIPPV in a resource poor setting or when invasive ventilator is unavailable during high demand situation particularly in selected patient with severe H1N1 influenza pneumonia.

Nevertheless, it is too premature to conclude that NIPPV is the modality of choice for treatment of acute respiratory failure due to H1N1 infection. It is also imprudent for us to await scientific evidence for taking clinical decisions. In the scenario of scarcity for invasive ventilators during such infectious outbreak, the expeditious use of noninvasive ventilation would help conserving the equipment for those who need it most.
